# Use of Orthodontic Methods in the Treatment of Dental Luxations: A Scoping Review

**DOI:** 10.3390/dj9020018

**Published:** 2021-02-04

**Authors:** Enrico Spinas, Laura Pipi, Silvia Mezzena, Luca Giannetti

**Affiliations:** 1 Department of Surgical Sciences, Sports Dental Research Center, University of Cagliari, Via Ospedale, 40-09124 Cagliari, Italy; enricospinas@tiscali.it; 2 Department of Conservative Dentistry and Endodontics, School of Dentistry, University of Cagliari, Via Ospedale, 40-09124 Cagliari, Italy; silvia@studio-mezzena.it; 3 Department of Surgery, Medicine, Dentistry and Morphological Sciences with Transplant Surgery, Oncology and Regenerative Medicine Relevance, University of Modena and Reggio Emilia, Via del Pozzo, 71-41124 Modena, Italy; luca.giannetti@unimore.it

**Keywords:** splinting technique, luxation injuries, physiological healing, pulp necrosis, root development, permanent teeth

## Abstract

(1) Background: Treating dental luxation injuries is challenging for the clinician. Dental luxations account for 18–33% of injuries to permanent teeth and can be addressed using different therapeutic approaches. The present work was conducted with two aims: (i) to evaluate, through a scoping review, current knowledge of the orthodontic methods (repositioning and stabilization splinting) that can be used at the time of the trauma, and (ii) to investigate the frequency and type of pulp consequences arising after these traumatic injuries. (2) Methods: The literature search was conducted in the period June 2020–December 2020 using the PubMed/MEDLINE, SCOPUS and Web of Science databases. The research questions were formulated according to the PICO (Population, Intervention, Comparison, Outcomes) method and considered the following aspects: type of luxation injury and stage of root development; use of orthodontic repositioning and splinting techniques; frequency and type of pulp consequences; and compliance of treatments with international guidelines. (3) Results: The initial screening of the databases, using the selected search keywords, yielded a total of 587 articles, just 8 fully met the inclusion criteria. Closer analysis of these 8 publications revealed that they would not produce clear meta-analytical data. This made it necessary to limit the data collected to the following six items: number and type of injuries, initial therapeutic intervention, duration of follow-up, number, and type of different pulp consequences. (4) Conclusions: While orthodontic techniques are commonly used to treat dental intrusions, in the case of extrusive and lateral luxation injuries, they are less frequently used and the orthodontic approach is generally confined to the stabilization phase. Among the various possible pulp consequences, many authors consider only pulp canal obliteration (PCO) and pulp necrosis (PN), often tending to overlook physiological healing (pulp survival) and the possible development of PN after PCO. There is therefore a clear need for new, high-quality clinical studies of this topic based on systematic and standardized data collection.

## 1. Introduction

Epidemiological studies show that 10.5–17.3% [1,2] of people suffer traumatic injuries to their teeth. These injuries can occur at any age, but youngsters aged 11–15 years are the group most frequently affected, accounting for around 13% of cases [3]. Luxation involving the pulp and/or supporting tissue constitutes a severe dental trauma and accounts for 18–33% [4,5] of injuries to permanent teeth. In dentistry, a luxation is a “displacement of a tooth from its original position in the alveolus, without total avulsion, resulting from acute trauma” [6] and it can be intrusive, extrusive, or lateral.

An intrusive luxation, or dental intrusion, is defined as a dislocation of the tooth in an apical direction into the socket [7,8]. It is considered the most severe type of luxation injury. Clinically, the crown of the intruded tooth appears shorter than that of the contralateral tooth. The prognosis, often poor, is correlated with the stage of root development: the chances of survival are greater for teeth with an immature root system [9,10].

An extrusive luxation injury, or partial avulsion [8,11], results in almost total disruption of the periodontal ligament attachment and rupture of the apical neurovascular bundle [12], causing tooth mobility. Clinically, the tooth appears elongated and often displaced palatally [8] with the presence of occlusal interference.

A lateral luxation is an eccentric dislocation of the tooth in a non-axial direction [12]; the damage to the apical neurovascular bundle is further complicated by fracture of the labial bone and compression of the tissue in the apical and cervical area [13].

Faced with these injuries, the possible therapeutic options, according to the IADT guidelines (International Association of Dental Traumatology) [7], are: passive repositioning (in cases of intrusive luxation), manual repositioning (all luxation types), intentional replantation, orthodontic repositioning, and splinting of the luxated tooth (all luxation types).

Dental intrusions are treated according to different protocols depending on the stage of root development [7]. The options are: passive repositioning (where the tooth is simply allowed to re-erupt), orthodontic repositioning, and surgical repositioning.

In extrusive luxation injury, if acute treatment is possible (i.e., within 3 h of the trauma), this will consist in manual repositioning of the extruded tooth followed by the placement of a passive and flexible stabilization splint. When this procedure is impossible, the clinician’s options are surgical or orthodontic repositioning of the extruded tooth. Intentional replantation (surgical repositioning) [14] involves extraction of the extruded tooth, which is then reinserted into the socket as quickly as possible [15]. This procedure can lead to inflammatory root resorption, external root resorption, or replacement resorption [16,17,18,19,20,21,22,23,24]. Indeed, the method has several contraindications and should therefore be used only in selected cases [18]. A promising alternative is orthodontic repositioning [25,26,27,28,29], which involves moving the tooth back into its original position. This is a slow, gradual process that takes 40/60 days on average and is followed by stabilization with a splint [26].

The treatment of lateral luxations involves manual repositioning of the tooth, after first disengaging it from the cortical bone; this is followed by a 4-week stabilization splinting period [7,30,31].

Dental splinting is crucial following a luxation injury and the IADT guidelines recommend the use of a passive and flexible splint [7]. However, various studies have shown that neither the type of splint nor the duration of stabilization significantly affects the healing outcome [32,33], even though comparison with non-splinted teeth has demonstrated that use of the technique can reduce stresses on traumatized teeth [34].

The possible pulp consequences of these dental luxation injuries include pulp canal obliteration (PCO), pulp necrosis (PN) and physiological healing (PS) [10,35]. The prevalence rates of these responses depend on the severity of the injury and the stage of root development of the injured tooth. PCO has been reported in 3–24% of cases after a luxation injury [10]; PN has been reported in 64% and 77% of cases after extrusive and lateral luxation, respectively, and in up to 100% following avulsion and intrusive luxation [35,36]; while physiological healing has been reported in approximately 20% of the luxated teeth [10].

In the literature, few cases of luxated teeth treated with orthodontic repositioning are reported and data are scare. According to Field and Christensen in 2013 [37], a laterally luxated tooth can be moved immediately after a trauma, using an orthodontics appliance with NiTi wire that generates light forces, and that it can be completely repositioned into the socket in about 3–5 days, although it may take longer. Similarly, there are only few reports [27,28,29] on the effectiveness of post-traumatic orthodontic repositioning (immediate or delayed) of extruded teeth and their survival over time. From these considerations, it emerges that orthodontic repositioning still presents a marginal role in the treatment guidelines of dental luxations.

This work was undertaken with a precise aim: to conduct a scoping review of the scientific literature in order to ascertain current knowledge on the orthodontic techniques (repositioning and stabilization splinting) that can be used by clinicians in patients who have suffered luxation injuries to the teeth. We examined the pulp consequences of these traumatic events and their distribution between teeth with open apex (OA) versus closed apex (CA); we also analyzed the frequency with which traumatized teeth showed different outcomes: PCO, PN, physiological healing (pulp survival), and PN after PCO.

## 2. Materials and Methods

We performed a scoping review of the literature and, on the basis of the data collected, highlighted the need to facilitate meta-analyses.

The review was conducted using the method “Scoping Review” described by Arksey and O’Malley [38], and also recently discussed by Zachary Munn et al. [39]. Essentially, it consists of 5 stages: identifying the research question; identifying relevant studies; study selection; charting the data; and collating, summarizing, and reporting the results.

In accordance with the 2015 PRISMA (Preferred Reporting Items for. Systematic Reviews and Meta-Analyses) guidelines [40], a work protocol was drawn up, setting out the steps in the literature review process: (1) research design and formulation of the questions; (2) selection of the keywords for the database searches; (3) definition of the inclusion and exclusion criteria; (4) literature search and listing of bibliographic citations deemed pertinent; (5) study selection process; (6) charting the data; (7) selected data collation and summarizing; and finally (8) discussion of the results drawn from the included articles.

### 2.1. Research Questions

The research focused on the following aspects: (1) the type of luxation injury sustained (intrusive, extrusive, or lateral) and the stage of root development at the time of the injury (considering teeth with OA versus teeth with CA); (2) the use of orthodontic repositioning and stabilization splinting techniques; (3) the frequency and type of pulp consequences (i.e., PCO, PN, pulp survival, or PN after PCO); (4) compliance with the IADT guidelines.

### 2.2. Selection Criteria

The search strategy was based on the PICO method [41], as follows:

- Population. We considered studies in humans presenting permanent dentition and one or more teeth affected by intrusive, extrusive, or lateral luxation injuries.
- Intervention. We considered patients treated with orthodontic repositioning and stabilization splinting, with the use of flexible, semi-rigid, or rigid splints.
- Comparison. We compared patients presenting with intrusive, extrusive, and lateral luxation injuries.
- Outcomes. We considered the number of cases of PCO and of PN in teeth affected by intrusive, extrusive, and lateral luxations; we also considered the number of teeth that showed physiological healing (pulp survival) and the appearance of PN after PCO in the three types of trauma.

We selected articles written in English and published after 2000. Included studies could be randomized clinical trials, observational studies (with cohort, case-control, or cross-sectional designs), clinical case series, or case reports. Reviews, in vitro, or animal studies, editorials, conference abstracts, letters, and comments were excluded.

**Figure 1:**
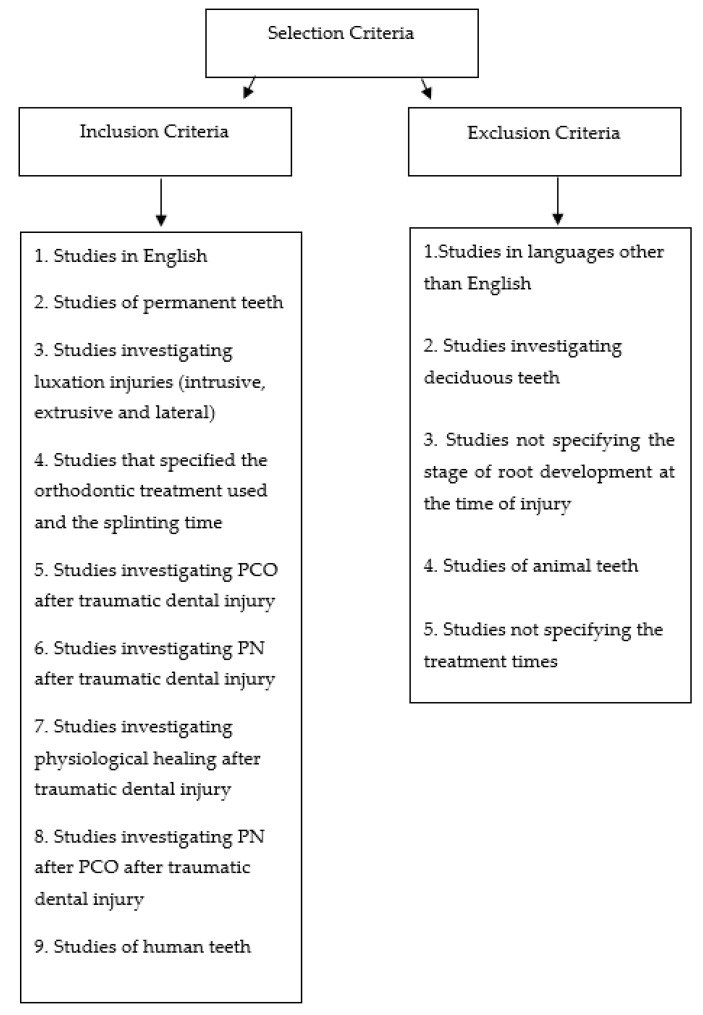
The figure contains either inclusion and exclusion criteria formulated for research.

The inclusion criteria are summarized in Figure 1.

### 2.3. The Search Strategy

The search was carried out during the period June 2020 to December 2020, using the following databases: PubMed/MEDLINE, SCOPUS, and Web of Science.

The following keywords were used: (1) Luxation Dental Injuries AND Permanent Tooth/Teeth, (2) Luxation Dental Injuries AND Splint, (3) Extrusive Luxation AND Obliteration, (4) Lateral Luxation AND Splint, (5) Intrusive luxation AND Resorption, (6) Pulp Canal Obliteration AND Luxation Injuries.

Two reviewers (LP and SM), working independently, selected relevant studies by title/abstract or, if these elements were inadequate, by reading the full text. The two reviewers then met to finalize the selection. Any disagreements were resolved by a third reviewer (ES), who decided whether or not to include the article in question.

### 2.4. Study Quality Assessment

The three reviewers (LP, SM, and ES) independently assessed the quality of the pre-selected studies. The kappa coefficient was used to evaluate the level of agreement between them, and it was found to be “moderate” [42]. Any disagreements were resolved through discussion and efforts to find a solution deemed valid by all three reviewers.

### 2.5. Data Extraction

Two reviewers (LP and SM) extracted the following data from each article: the publication date (year), the first name in the list of authors, the type of luxation injury (intrusive, extrusive, or lateral), the stage of dental root development at the time of the trauma (OA or CA), the use of orthodontic repositioning and splinting techniques, the main pulp consequences (i.e., the presence of PCO, PN, physiological healing, PN after PCO).

Quality control of the studies was carried out by calculating the risk of bias, in accordance with the criteria recommended in the “Cochrane Handbook for Systematic Review of Interventions, version 5.1.0” [43]. The value obtained was incompatible with a systematic review, but not with a scoping review.

Cohen’s kappa test was run to evaluate the level of agreement between the two reviewers, and the value (0.61) corresponded to “moderate”.

The data extracted from the included studies were entered into a Microsoft Excel 2016 spreadsheet for analysis (Table 1 and Table 2).

## 3. Results

**Figure 2:**
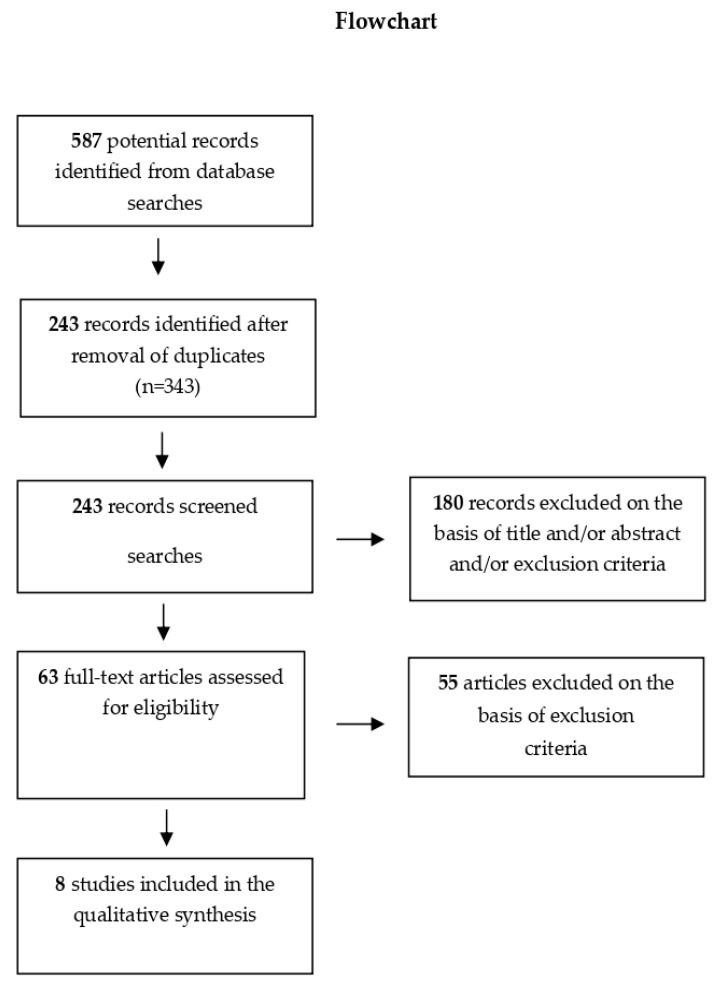
Flowchart illustrating the search and selection strategy used to identify the included articles.

The initial search, based on review of titles and abstracts, yielded a total of 587 potentially relevant articles (Figure 2).

After closer screening, 343 were eliminated because they were found to be duplicates. Of the remaining 243 articles, a further 180 were eliminated on the basis of title and/or abstract and/or exclusion criteria. Only 63 were deemed to warrant reading of the full test, to assess their eligibility. On full-text reading, 8 articles were found to be completely in line with our inclusion criteria. Even though 13 articles reported the treatments used, only the final 8 specified the types of equipment used, the splinting times, and the stage of root development at the time of the trauma. This latter aspect was crucial to our study, which aimed to explore whether it could determine differences in pulp consequences manifested during follow-up.

Table 3 lists the main features of the eight studies included in the review (first listed author, publication year, title, and type of study), while Table 4 summarizes, according to the lesion type and type of orthodontic treatment used (repositioning or stabilization), the information on pulp responses reported in these studies.

## 4. Discussion

In the treatment of intrusive luxation injuries to the teeth, orthodontic extrusion is a widely discussed and well proven method, which has become part of daily practice [7]. Conversely, the literature contains very little information on the use of the orthodontic repositioning technique in extrusive and lateral luxations.

Although few authors use orthodontic repositioning, the technique can prove useful when manual repositioning of the dislocated tooth is impossible. In reports of orthodontic repositioning, the intervention was mostly delayed, taking place days or weeks after the traumatic event and often involving a necrotic tooth [27,28,29,44].

The benefits of orthodontic repositioning are linked to three key factors: (a) the application of extremely light and controlled forces; (b) the tissue reorganization permitted by the application of light forces [26,28]; and (c) the fact that there is no need for local anesthesia. This latter aspect, albeit often overlooked, is of considerable importance when dealing with a child who is already frightened and upset [37].

As mentioned above, orthodontic repositioning could replace manual repositioning of the tooth when the latter cannot be performed and this is an important consideration. Furthermore, during both manual and surgical repositioning, the force applied to the tooth cannot be fully controlled, with the result that these techniques can potentially inflict a further trauma on the already damaged tooth [28].

Orthodontic appliances are less invasive nowadays and since they apply light, gradual, and controlled forces, they are less likely to further damage the apical neurovascular supply [28,37,45].

The splint should be bonded to the two healthy teeth adjacent to the luxated tooth. No extra benefit has been found when using larger splints, involving a greater number of teeth [46].

With regard to the choice of splint, in all the papers considered, the IADT guidelines had been followed. The following types were used: flexible/elastic (Kevlar splint and elastic orthodontic wire, TTS composite, wire-composite) or semi-rigid (stainless steel 0.014/0.016 wire composite, 0.9 mm fishing line, bracket, and NiTi wire). The wire-composite splint was the type most frequently used.

The purpose of stabilization splinting is to keep the tooth stable for as long as is necessary to avoid further damage and protect the dental attachment apparatus, giving the periodontal fibers chance to regenerate [7,47].

The duration of stabilization depends on whether the tooth was repositioned surgically or manually. The IADT guidelines recommend a period of 4–8 weeks after a dental intrusion, 2 weeks after an extrusive luxation and 4 weeks after a lateral luxation. In an extrusive or lateral luxation, the splinting time can be increased by a further 4 weeks if there is breakdown/fracture of the marginal bone [7].

Our review revealed that only a few of the authors applied the guidelines from the early diagnostic stages. Furthermore, few specified the severity of the sustained injuries, the time between the traumatic event and the patient’s access to treatment and the various clinical and radiographic investigations carried out at the time of the trauma, even though all these are important parameters [7,48,49].

These information gaps make it impossible to understand the choices made by clinicians; furthermore, in cases where treatment was delayed, the reason for this was often not given (e.g., Was it a clinical decision to wait for the tooth to re-erupt spontaneously? Or was it the patient who sought treatment late?). Information on treatment delay is important, as this aspect can statistically influence both the pulp consequences observed [9,26] and the repositioning methods that can be used [50].

**Figure 3:**
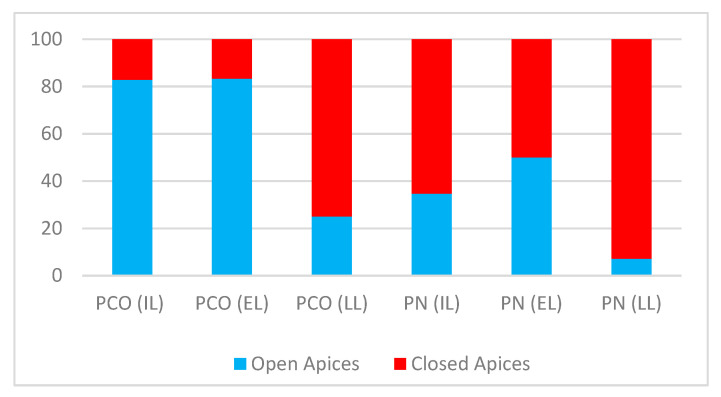
The figure shows the percentage rates of pulp necrosis (PN) and pulp canal obliteration (PCO) in intrusive luxation (IL), extrusive luxation (EL), and lateral luxation (LL).

Among the studies we reviewed, with regard to the pulp consequences (see Figure 3), of the 111 teeth with intrusive luxation [51,52], 87 showed signs of PCO or PN after the dental trauma. In detail, PCO was diagnosed in 35/111 teeth (29 with OA and 6 with CA), while PN was found in 52/111 (18 with OA and 34 with CA) [Table 4].

Of the 72 teeth showing extrusive luxation [26,53,54,55], 58 showed pulp responses after the trauma: 30/72 showed PCO (25 with OA and 5 with CA), while PN was diagnosed in 28/72 teeth (14 with OA and 14 with CA) [Table 4].

Finally, of the 105 laterally luxated teeth [30,31], 74 showed pulp responses. In detail, PCO was diagnosed in 32/72 teeth (8 with OA and 24 with CA), while PN was diagnosed in 42 (3 with OA and 39 with CA) [Table 4].

From a descriptive analysis of the data, PN emerged as the most frequent pulp response both after intrusive (52/111 teeth) and lateral luxations (42/105). In both types of injury, PN was far more likely to occur in the presence of closed apex (34/52 and 39/42, respectively) as opposed to open apex (18/52 and 3/42). These results are justified by the biology of the damage, given that both these types of injury carry an increased risk of damage to the periodontal ligament and dental pulp [8,10,13,56].

In our study, the higher incidence of PCO in laterally luxated teeth with closed apex is the only finding that seems to conflict with literature data [10,57,58]. Among the laterally luxated teeth (*n* = 105), 32 elements showed PCO and of these only 25% had open apices (8/32) while 75% had closed apices (24/32).

The present study mainly considered young patients, aged between 6 and 18 years; only Ferrazzini Pozzi’s [31] sample had a broader age range (7–59 years).

For the sake of completeness, analysis of the pulp consequences of dental luxations should also consider cases of pulp survival (physiological healing) and cases of PN in teeth already showing PCO.

Although, in the literature, the evaluation of pulp survival dates to 1987 [10], few authors refer to this post-traumatic outcome and the topic seems to be the subject of some confusion. Among the studies we reviewed, those of Tsilingaridis [52] and Ferrazzini Pozzi [31] report pulp survival in 17/60 and 19/47 cases, respectively. However, the clinical basis of these diagnoses is unclear.

The doubt lies in the fact that a tooth, to be considered physiologically healed, must show complete root development, vital pulp (retain pulp sensitivity), and no clinical or radiographic abnormalities [10,26].

Similarly, not all the studies took into consideration cases of PN developing after a diagnosis of PCO. In those that did explore this aspect, this event was found to be very rare: 2/55 teeth in Lee [53]; and absent in the reports by Spinas [26], Ramirez [54], Cehreli [55] and Nikoui [30]. This reflects what is found in the literature, in which the rate of PN after PCO ranges from 1 to 27%, and it is, indeed, considered a very rare occurrence [59]. The onset of PN in teeth already diagnosed with PCO may be linked to disruption of the dental pulp vascularization, due, for example, to orthodontic tooth movement (particularly intrusion); similarly, new traumas, if associated with a narrowed pulp canal space, can increase the risk of PN [60,61,62,63].

Various authors maintain that endodontic treatment should be performed only in cases of primary PN and that the diagnosis should be based on negative pulp sensitivity tests in the presence of clear clinical and radiographic findings [58,59,64,65].

Ultimately, the findings of this scoping review prompt several interesting clinical considerations:

(1) The stabilization splinting technique is a fundamental part of the management of luxated teeth that have undergone dental repositioning, regardless of the dental movement method used. Orthodontic repositioning of an intruded tooth is, in fact, a treatment approach that has been widely validated in the literature and it can also be considered, in certain situations, for the treatment of extrusively and laterally luxated teeth. However, even though it does not seem to aggravate pathological pulp responses in these cases and does not necessarily have to applied immediately after the trauma, orthodontic repositioning is rarely considered in the treatment of these injuries. Further observational and retrospective studies are needed to validate this protocol.
(2) Compliance with the guidelines in the early diagnostic and follow-up phases, and in relation to splinting times, was found to be poor. The lack of data on the timing of treatments and the severity of the luxation (e.g., mild, moderate, and severe), which to an extent justifies the choice of treatment, did not allow us to obtain conclusive information.
(3) Pulp necrosis is the most frequent pathological pulp reaction in teeth that have sustained an intrusive or lateral luxation injury, showing a higher prevalence, of approximately 66% and 93% respectively, in CA teeth.
(4) Pulp canal obliteration is a physiological response to luxation injuries, particularly dental extrusions (in which it is seen in around 42% of cases); it occurs mainly in OA teeth (approximately 83%).
(5) The literature confirmed that the appearance of PN in a tooth showing PCO is a rare occurrence. Furthermore, since physiological healing (pulp survival) is always one of the possible outcomes, it should be more extensively investigated by clinicians. The present review showed that this outcome was not reported by all authors, and even when it was reported, the information provided was unclear.

## 5. Conclusions

The current literature on use of the orthodontic dental repositioning technique after luxation injuries appears controversial. The lack of standardized data collection and the difficulty in achieving precise and detailed superimposition of clinical data makes it impossible to provide clear clinical indications in a field that therefore demands further research.

The results of the present scoping review highlight the need for further clinical studies based on systematic and standardized data collection [48,49]. This will allow homogenization of the data, which in turn, will facilitate meta-analyses and allow more definitive therapeutic information to be drawn from investigations of this topic.

## Figures and Tables

**Table 1:** The table shows data from the included studies, summarizing diagnostic and clinical information.

Author and Year	N° of Samples	N° of Intr. Lux.	N° of Extr. Lux.	N° of Lat. Lux	Mm of lux	PCO in IL	PCO in EL	PCO in LL	PN in IL	PN in EL	PN in LL	PS	PN after PCO	First Clinical Examination	Follow-Up
Lee, 2003	55	/	55 (24 OA 31 CA)	/	<2 mm >2 mm n° NS	/	19 (16 OA 3 CA)	/	/	23 (11 OA 12 CA)	/	NS	2	Clinical examination, X-ray (Periapical+ Occlusal)	2 wk-3 mo-6 mo-early
Nikoui, 2003	58	/	/	58 (35 OA 23 CA)	<2 mm >2 mm n° NS	/	/	23 (6 OA 17 CA)	/	/	23 (3 OA 20 CA)	NS	0	Clinical examination, X-ray (Periapical+ Occlusal)	2 wk-3 mo-6 mo-early
Wigen, 2008	51	51 (31 OA 20 CA)	/	/	<2 mm (8) >2 mm (22)	18 (13 OA 5 CA)	/	/	29 (14 OA 15 CA)	/	/	4	NS	Clinical examination, X-ray (Periapical)	1–12 yr
Tsilingaridis, 2011	60	60 (27 OA 33 CA)	/	/	1–3 mm (16) 4–6 mm (22) >7 mm (22)	17 (16 OA 1 CA)	/	/	23 (4 OA 19 CA)	/	/	17	NS	Clinical examination, X-ray (Periapical)	6–130 mo
Cehreli, 2012	2	/	2 (2 OA)	/	NS	/	/	/	/	2 (2 OA)	/	0	0	Clinical examination, X-ray (Periapical)	1–2-3 wk- 3 mo-12 mo-18 mo
Ferrazzini Pozzi, 2008	47	/	/	47 (10 OA 37 CA)	NS	/	/	9 (2 OA 7 CA)	/	/	19 (0 OA 19 CA)	19	NS	Clinical examination, DPT, X-ray	2 wk- 4 wk- 6/8 wk-6 mo-directly at 4 yr
Ramirez, 2018	2	/	2 (2 OA)	/	NS	/	2 (2 OA)	/	/	0	/	0	0	Clinical examination, X-ray (periapical), photo	2 wk-3-4-5 mo-1–3-4 yr
Spinas, 2020	13	/	13 (8 OA 5 CA)	/	0–2 mm (4) 3–5 mm (7) >6 mm (2)	/	9 (7 OA 2 CA)	/	/	3 (1 OA 2 CA)	/	1	0	Clinical examination, DPT, X-ray (Periapical+ Occlusal), photo	2 wk- 4 wk- 6/8 wk-6 mo-yearly for 5 yr

Legend: PCO = pulp canal obliteration; PN = pulp necrosis; PS = physiological healing; IL = intrusive luxation; EL = extrusive luxation; LL = lateral luxation; wk = weeks; mo = months; yr = year; OA = open apices; CA = closed apices; NS = not specified; n° NS: Number of teeth not specified.

**Table 2:** The table shows the type of treatment choice, materials, and time of use.

Author and Year	N° of Samples	Age (Years)	N° of Intr. Lux.	N° of Extr. Lux.	N° of Lat. Lux	Manual Repositioning	Surgical Repositioning	Orthodontic Repositioning	Time of Splinting	Type of Splinting
Lee, 2003	55	7.1–17.8	/	55 (24 OA 31 CA)	/	55	0	0	7–14 days	SS 0.016 wire composite
Nikoui, 2003	58	6.3–17.8	/	/	58 (35 OA 23 CA)	58	0	0	14–21 days	SS 0.014/0.016 wire composite
Wigen, 2008	51	6–17	51 (31 OA 20 CA)	/	/	37	7	7	2–6 wk	Wire composite
Tsilingaridis, 2011	60	6–16	60 (27 OA 33 CA)	/	/	17	12	31	6–80 days (mean 28.9)	Kevlar + wire
Cehreli, 2012	2	8.5	/	2 (2 OA)	/	2	0	0	3 wk	Fishing line + composite
Ferrazzini Pozzi, 2008	47	7–59	/	/	47 (10 OA 37 CA)	47	0	0	7–28 days (mean 22)	TTS composite
Ramirez, 2018	2	9	/	2 (2 OA)	/	2	0	0	3 mo	SS wire composite 0.4 mm
Spinas, 2020	13	8–16	/	13 (8 OA 5 CA)	/	3	9	0	14–21 days	Bracket–NiTi wire

Legend: OA = Open Apices; CA = Closed Apices; wk = Weeks; mo = Months.

**Table 3:** The table summarizes the main characteristics of the studies included in the review.

Author, Years	Title	Type of Study
Lee, 2003	Clinical outcomes for permanent incisor luxations in a pediatric population. II. Extrusion	Longitudinal study
Nikoui, 2003	Clinical outcomes for permanent incisor luxations in a pediatric population. III. Lateral Luxations	Longitudinal study
Wigen, 2008	Intrusive luxation of permanent incisors in Norwegians aged 6–17 years: a retrospective study of treatment and outcome	Retrospective study
Ferrazzini Pozzi, 2008	Pulp and periodontal healing of laterally luxated permanent teeth: results after 4 years	Retrospective study
Tsilingaridis, 2011	Intrusive luxation of 60 permanent incisors: a retrospective study of treatment and outcome	Retrospective study
Cehreli, 2012	Revascularization of Immature Permanent Incisors after Severe Extrusive Luxation Injury	Case report
Ramirez, 2018	A 4-year follow-up case of extrusive luxation in a patient with cerebral palsy	Case report
Spinas, 2020	Extrusive luxations in young patients: Retrospective study with 5-year follow-up	Retrospective study

**Table 4:** The table shows the pulp responses after luxation injuries and the therapeutic approach used.

*Intrusive Luxation*				
Total	PCO	PN	PS	PN after PCO
111 58 OA–53 CA	35 29 OA–6 CA	52 18 OA–34 CA	19	-
*Extrusive Luxation*				
Total	PCO	PN	PS	PN after PCO
72 36 OA–36 CA	30 25 OA–5 CA	28 14 OA–14 CA	1	2
*Lateral Luxation*				
Total	PCO	PN	PS	PN after PCO
105 45 OA–60 CA	32 8 OA–24 CA	42 3 OA–39 CA	19	0
*Intrusive Luxation*				
Total	Manual repositioning	Orthodontic repositioning	Surgical repositioning	
111 58 OA–53 CA	54	19	38	
*Extrusive Luxation*				
Total	Manual repositioning	Orthodontic repositioning	Surgical repositioning	
72 36 OA–36 CA	62	12	0	
*Lateral Luxation*				
Total	Manual repositioning	Orthodontic repositioning	Surgical repositioning	
105 45 OA–60 CA	105	0	0	
